# Chitosan-Based Bioactive Glass Gauze: Microstructural Properties, In Vitro Bioactivity, and Biological Tests

**DOI:** 10.3390/ma13122819

**Published:** 2020-06-23

**Authors:** Rachele Sergi, Devis Bellucci, Roberta Salvatori, Valeria Cannillo

**Affiliations:** 1 Dipartimento di Ingegneria Enzo Ferrari, Università degli Studi di Modena e Reggio Emilia, Via P. Vivarelli 10, 41125 Modena, Italy; rachele.sergi@unimore.it (R.S.); devis.bellucci@unimore.it (D.B.); 2 Laboratorio dei Biomateriali, Dipartimento di Scienze Mediche Chirurgiche Materno-Infantili e dell’Adulto, Università di Modena e Reggio Emilia, Via Campi 213/A, 41125 Modena, Italy; roberta.salvatori@unimore.it

**Keywords:** chitosan/bioactive glass wound dressings, cytotoxicity, SBF, biological tests, scratch test

## Abstract

Passive commercial gauzes were turned into interactive wound dressings by impregnating them with a chitosan suspension. To further improve healing, and cell adhesion and proliferation, chitosan/bioactive glass wound dressings were produced with the addition of (i) 45S5, (ii) a Sr- and Mg-containing bioactive glass, and (iii) a Zn-containing bioactive glass to the chitosan suspension. SEM and FTIR analyses evidenced positive results in terms of incorporation of bioactive glass particles. Bioactivity was investigated by soaking chitosan-based bioactive glass wound dressings in simulated body fluid (SBF). Cell viability, proliferation, and morphology were investigated using NIH 3T3 (mouse embryonic fibroblast) cells by neutral red (NR) uptake and MTT assays. Furthermore, the wound-healing rate was evaluated by means of the scratch test, using NIH 3T3. The results showed that bioactive glass particles enhance cell adhesion and proliferation, and wound healing compared to pure chitosan. Therefore, chitosan-based bioactive glass wound dressings combine the properties of the organic matrix with the specific biological characteristics of bioactive glasses to achieve chitosan composites suitable for healing devices.

## 1. Introduction

In recent years, with the increase in diabetic problems, scientists have been exploring different materials for the treatment of wounds from natural and synthetic sources [1]. An ideal wound dressing should maintain wound moisture, protect wounds from environmental discomfort and bacteria, adsorb exudates, and enhance healing. Thus, wound dressings should be non-toxic and non-allergenic with antibacterial and hemostatic properties. In addition, an ideal wound dressing should also be readily available, economic, and should be removed without pain for the patient.

The regeneration of wound tissue is a complicated process that involves blood clotting, epithelialization, wound contraction, and synthesis of collagen and blood vessels [2,3,4,5]. The healing process comprises a precise sequence of harmonized steps in which migratory and resident cell populations, components of the extracellular matrix, and soluble mediators are involved [6].

Disparate wound types go through a sequence of mechanisms: 1) Hemostasis, in which a fibrin clot is formed to prevent blood loss through vasoconstriction, as well as prevent microbial contamination [7]; (2) inflammatory phase, which begins almost simultaneously with hemostasis. Inflammatory cells move from surrounding tissues to the injured sites, resulting in the formation of fibroblasts to produce collagen; connective collagen provides tensile strength to the new tissue. (3) and (4) include the process of neovascularization, which starts from capillaries formation and provides nutrients and oxygen at the site and cell migration. Epithelial cells around the wound fill the zone under the scab. Fibroblasts migrate to the wound site and differentiate into myofibroblasts to generate components of the extracellular matrix such as collagen, fibronectin, hyaluronic acid, and proteoglycan, which take part in re-epithelialization and new blood vessels [7]. (5) In the final phase, maturation and remodeling, the synthesis of the new epithelium completes the wound healing process [8,9] and all steps that were activated after injuries are interrupted [10]. Therefore, wound healing is considered one of the most complicated processes that take place in the human body. Up to now, different types of wound dressings like hydrogels, films, membranes, sponges, and electrospun mats have been developed to improve the healing process. In order to guarantee a successful wound healing process, a controlled set of conditions (i.e., temperature, minerals, oxygenation, high viability of vitamins) at the wound site should be maintained to sustain the complex cellular activities during the process [11]. The incorporation of molecules (antibiotics, vitamins, ions, anti-inflammatory molecules, molecules from natural extracts, grow factors—GFs) into wound dressings has been studied to avoid skin infection and mediate the various phases of healing to a more successful skin regeneration. Despite improving results in skin regeneration, there are still several challenges for the incorporation of molecules into healthcare applications. Nevertheless, the realization of wound dressings is fundamental to contribute to the enhancement of the quality of life of patients. To fabricate wound dressings, several biocompatible polymers—both natural and synthetic—have been employed; among them, chitosan can be a promising natural polymer for wound dressing applications [1,12,13]. Chitosan (CHT) is non-toxic and biocompatible and its use is increasing in medical and pharmaceutical applications.

CHT is a linear polysaccharide composed of N-acetyl-d-glucosamine (acetylated unit) and β-(1-4)-linked d-glucosamine (deacetylated unit) randomly distributed. It is produced by the deacetylation of shrimps and other crustacean shells with the alkali NaOH to eliminate the acetamide group. The conversion to the amine group is never absolute; it varies from 30% to 95%. The deacetylation degree (DD) influences the biocompatibility, which decreases with increasing DD, solubility, and degradation rate. Furthermore, CHT presents antimicrobial, hemostatic, and analgesic properties, stimulating closure, neovascularization, and permanent regeneration of the dermis in the wound [14]. The cationic behavior and the size of the polymer chain influence the biological properties of chitosan. For instance, for its antimicrobial properties, two different mechanisms are proposed: one is based on its negative charges, which interact with the membranes of bacteria, affecting the permeability; the other is based on the capacity of chitosan to bond DNA of bacteria, avoiding RNA synthesis [15]. Both mechanisms lead to bacteria death. Despite these interesting properties, chitosan generally has low bioactivity and degradability; thus, bioactive glasses have been added to further improve cell adhesion [16,17]. In addition, gauzes with bioactive glass particles and nanoparticles have been successfully prepared [18,19,20].

In this work, novel chitosan-based bioactive glass wound dressings were developed starting from commercial gauzes; such commercial gauzes are simply inactive devices that safeguard the wound from other lesions while wound healing occurs naturally. Impregnating commercial gauzes with the chitosan suspension has the aim to transform passive gauzes into interactive products, which can change the local environment of wounds [21]. For this reason, commercial gauzes were soaked into the chitosan suspension with the aim to develop hemostatic wound dressings [22,23]. Furthermore, the addition of different amounts (5 and 10 wt.%) of bioactive glasses (45S5, BGMS10 [24] and BGMS_2Zn [25], respectively) introduces high bioactivity and antimicrobial effects to chitosan wound dressings. Indeed, bioactive glasses enhance the bioactivity and biodegradability of chitosan as reported in the literature [26,27]. BGMS10 and BGMS_2Zn have been shown to enhance cell adhesion and proliferation and have antimicrobial effects, respectively [24,25,28,29,30,31]. Sr and Mg ions are widely known to improve the replication of preosteoblastic cells [32,33,34]; however, such ions stimulate cell proliferation and angiogenesis as well [35,36,37]. On the other hand, Zn is essential for cell growth and proliferation [38,39], but it also takes part in enzyme production, replication of DNA, and GFs [40]. Additionally, Zn ions have shown antimicrobial effects [41,42] that can improve the antimicrobial properties of chitosan [43]. Therefore, the addition of bioactive glasses can result in improving wound healing, because bioactive glasses (i) release ions that stimulate a specific cellular response and (ii) may favor antimicrobial effects. Thus, a combination of chitosan and bioactive glasses could bring the advantages of both materials to improve wound healing.

## 2. Materials and Methods

### 2.1. Preparation of Glass Powders

The bioactive glasses were produced by a classical melt-quenching route, according to the process reported for other bioactive glasses [44,45,46,47]. The compositions of BGMS10 [24] and BGMS_2Zn [25] are shown in Table 1, along with the composition of 45S5 (Bioglass^®^) [48].

Briefly, the commercial raw powders (SiO_2_, CaCO_3_, Ca_3_(PO_4_)_2_, Na_2_CO_3_, K_2_CO_3_, SrCO_3_, MgCO_3_, ZnO, all reagent grade, Carlo Erba Reagenti, Milano, Italy) were mixed for 3 h in a laboratory shaker (M63a4-Motori Elettrici Carpanelli, Bologna, Italy) and then melted in a Pt crucible in air. The thermal cycle was: (i) From room temperature to 1100 °C at 10 °C/min; (ii) decarbonation step at 1100 °C for 2 h; (iii) from 1100 °C to 1450 °C at 10 °C/min; (iv) and 45 min at 1450 °C. The molten glasses were quenched in room-temperature water to obtain a frit, and the frit of each glass composition was then dried at 110 °C. After a night in the oven, the frit was milled in dry conditions in a porcelain jar to obtain powders with a final grain size lower than 38 μm. Finally, the bioactive glass powders were incorporated into chitosan solution to obtain chitosan-based bioactive glass wound dressings.

### 2.2. Chitosan-Based Bioactive Glass Wound Dressings Preparation

Wound dressings (2 cm × 2 cm squared shape) were obtained by soaking commercial gauzes (DERMATESS PLUS, MASTER-AID^®^, Pietrasanta Pharma S.p.A, Capannori, Italy) for 1 h in a chitosan solution. The amounts of materials used to prepare chitosan-based bioactive glass wound dressings are summarized in Table 2.

For the preparation of the control gauze of chitosan, 1 wt.%/Vtot medium-molecular-weight chitosan (Sigma-Aldrich, Darmstadt, Germany, medium molecular weight) was dissolved in an aqueous acetic acid solution 2 wt.%/Vtot under stirring for 5 min at 32 °C. Then, a commercial gauze was immersed for 1 h in such chitosan suspension at room temperature without stirring and then dried for 24 h at 36 °C. For chitosan-based bioactive glass wound dressings, the same chitosan suspension (1 wt.%/Vtot chitosan, 2 wt.%/Vtot acetic acid) was prepared. Subsequently, different amounts of bioactive glasses were added under stirring to different chitosan suspensions: 5 and 10 wt.% of 45S5, BGMS10 and BGMS_2Zn, respectively. Then, a commercial gauze was immersed for 1 h at room temperature without stirring and then dried for 24 h at 36 °C.

### 2.3. Microstructural Characterization

The surfaces of the chitosan wound dressing (CHT) and chitosan-based bioactive glass wound dressings (CHT45S5_5, CHT45S5_10, CHTSrMg5, CHTSrMg10, CHTZn5, and CHTZn10) were studied by Environmental Scanning Electron Microscopy (ESEM Quanta 2000, FEI Co., Eindhoven, the Netherland) before and after immersing samples in simulated body fluid (SBF) solution (see Section 2.5). Furthermore, local compositional analyses by energy-dispersive X-ray spectroscopy (EDS, Inca, Oxford Instruments, Abingdon, UK) were performed. Moreover, CHT, CHT45S5_5, CHT45S5_10, CHTSrMg5, CHTSrMg10, CHTZn5, and CHTZn10 wound dressings were investigated by means of FTIR (Nicolet 6700, Thermo Scientific, Schwerte, Germany). The spectra were detected between 400 and 4000 cm^−1^ with a resolution of 4 cm^−1^.

### 2.4. Swelling Tests

To measure the swelling behavior, wound dressings were cut into 1 cm × 1 cm pieces and their dry weights (D) were immediately measured; then, the dry wound dressings were soaked in distilled H_2_O at room temperature and their weights (I) were determined at 30 min, 1 h, 2 h, 3 h, 4 h, 1 day, 2 days, 3 days, and 4 days. The same experiment was repeated for 10 samples for each wound dressings’ composition, and the mean value was taken as the swelling ratio. The swelling ratio (SR%) was calculated as follows:

(1) $$\mathrm{SR}\%=\frac{I-D}{D}\times 100$$

where “I” is the samples’ weight after immersion at each time point, and “D” is the dry samples’ weight [49,50,51].

### 2.5. In Vitro Bioactivity

Wound dressings (CHT, CHT45S5_5, CHT45S5_10, CHTSrMg5, CHTSrMg10, CHTZn5, and CHTZn10) were soaked in simulated body fluid (SBF) solution, prepared following the protocol developed by Kokubo [52], to investigate the eventual formation of hydroxy-carbonate-apatite (HCA) on their surface once in contact with physiological fluids. The wound dressings were soaked in SBF for 1, 3, and 7 days; after soaking samples for the prescribed time, each of them was washed immediately with distilled water and dried at room temperature. The eventual HCA layer formation on the surface of samples was studied by ESEM.

### 2.6. Biological Tests

The in vitro biocompatibility of wound dressings was evaluated using NIH 3T3 (mouse embryonic fibroblast) cells. The NIH 3T3 cell line was provided by ATCC^®^ (Milan, Italy). The cytotoxicity of wound dressings was investigated according to International Standards ISO 10993: (i) 10993-1 for the selection of tests; (ii) 10993-12 for samples preparation and reference materials; and (iii) 10993-5 for the in vitro methods [53,54,55]. The direct contact test (neutral red (NR) uptake assay) and the indirect contact test (MTT assay) were used to assess cytotoxicity of the wound dressings. For the NR uptake assay, the incorporation of neutral red dye (NR dye) into cells permits the differentiation between viable, damaged, or dead cells, because any alteration in lysosomal membrane or change in the cell surface causes a decrease in NR dye binding. This decrease in the incorporation of NR dye results in a low value of optical density detected by the spectrophotometer (Multiscan RC by Thermolab system, Vantaa, Finland). For the MTT assay, tetrazolium salts are intracellularly reduced into formazan by mitochondrial succinate dehydrogenase enzyme (SDH) of viable cells (crystal of a blue-purple product). This reduction takes place in metabolically active cells only. Then, a solubilization solution (DMSO) is added to dissolve the formazan product into a colored solution. Finally, the formazan concentration accumulated into metabolically live cells is directly quantified using the spectrophotometer. Wound dressings were sterilized by UV irradiation for 1 h before starting the biological tests.

#### 2.6.1. Culture of NIH 3T3

Dulbecco’s modified Eagle’s Medium (DMEM) containing 1 mM sodium pyruvate, L-glutamine 2 mM, 10% (v/V) FBS (Fetal Bovine Serum-Invitrogen), and pen-streptomycin was used for growing NIH 3T3 cells. Cells were cultured in 12-well plates; the plates were maintained in an incubator at a temperature of 37 °C ± 1 °C, humidity of 90% ± 5%, and CO_2_/air ratio of 5% ± 1%. For each type of wound dressing, at least 3 replicates were prepared and tested at 24 and 72 h.

#### 2.6.2. Samples’ Eluates Preparation

CHT, CHT45S5_5, CHT45S5_10, CHTSrMg5, CHTSrMg10, CHTZn5, and CHTZn10 wound dressings were immersed in test tubes containing DMEM with a 3 cm^2^/mL ratio between standard surface and liquid volumes according to [54]. As a positive control (CTRL+), latex samples (Sensichlor powder free-Lot. 45126, Bericah S.p.a, Torri di Arcugnano, Italy) were used with the ratio 3 cm^2^/mL (surface of sample/liquid volume). DMEM only was used as the negative control (CTRL-) instead. The test tubes were incubated at a temperature of 37 °C for 72 h. Before testing, wound dressings’ eluates were filtered using a 0.22 μm filter (Millex-Gs 0.22 µm. Millipore Fr, Alsace, France).

#### 2.6.3. NR Uptake Assay at 24 and 72 h

NIH 3T3 were cultured in direct contact with wound dressings in 12-well plates at a temperature of 37 °C ± 1 °C, humidity of 90% ± 5%, and CO_2_/air ratio of 5% ± 1%. Then, 24 and 72 h after seeding, the morphology of cells was observed by an optical microscope (Leitz, Stuttgart, Germany). Then, DMEM was removed and 150 μL of NR solution was poured in each well. At the end of 3 h at 37 °C ± 1 °C of incubation, the NR solution was removed and 150 µL pre-warmed D-PBS was used to carefully wash cells, in order to remove the excess of NR solution. Subsequently, 1500 µL NR Desorb (i.e., the freshly prepared extract solution of ethanol/acid acetic) solution was added to each well, before incubating the 12-well plates at room temperature for 20 min, in order to extract NR dye from cells. Finally, the final solution was poured into 96 multi-well plates and the amount of NR dye bound by cells was investigated by a spectrophotometer at 540 nm absorbance.

#### 2.6.4. MTT Assay at 24 and 72 h

In 96 multi-well plates, NIH 3T3 cells (concentration of 5 × 10^4^/mL) were put in contact with 50 μL of eluates of wound dressings for 24 and 72 h. After each time point, 10 μL of tetrazolium salt (5 mg/mL in PBS) was poured into each well. Then, the media were carefully removed 3 h after incubation at 37 °C; 100 μL of DMSO was used to solubilize formazan. Finally, the absorbance of each well was measured at 540 nm to quantify the concentration of formazan synthesized inside cells.

#### 2.6.5. Scratch Test

The in vitro scratch test is an inexpensive, easy, and well-developed method to measure cell migration in vitro [56]. The scratch test is based on the observation of a gap (i.e., the “scratch”) on a confluent monolayer of cells until new cell–cell contacts are created again. Upon creation of the “scratch,” images at the beginning and at regular intervals during cell migration are taken. Finally, the images taken at each time point are correlated to determine the rate of cell migration. The scratch test mimics, to some extent, the migration of cells in vivo, and it is useful to evaluate the regulation of cell migration by the cell interaction with extracellular matrix (ECM) and cell–cell interaction [57]. Additionally, the scratch test is the simplest method to investigate the migration of cells in vitro and uses inexpensive and common supplies that could be easily found in most laboratories of cell culturing. On the other hand, the scratch test does not replace well-recognized method for chemotaxis and the chemical gradient is not established. Moreover, a relatively longer time and a large number of chemicals and cells are necessary for the scratch test. However, the scratch test is still the choice to analyze cell migration in vitro because it is easy to set up, and it does not require any specialized equipment [57]. NIH 3T3 cells were seeded in 24 multi-wells to create a confluent monolayer. Multi-wells were incubated at 37 °C, allowing cells to grow until the formation of a confluent monolayer. The number of cells for such a monolayer depends on the size of the multi-well and the type of cells. Subsequently, a 200 μL pipet tip was used to scrape the cell monolayer in a straight line, creating the “scratch.” Cells were rinsed with 500 µL of PBS, and 500 μL of medium (DMEM) and 500 μL of wound dressings’ eluates were then poured in each well. A mark close to the scratch was created as a reference point, in order to obtain the same field during the image acquisition. After taking the images at time 0, the multi-wells were incubated at 37 °C until the next time point. Images were taken at 3 time points: Time 0, time 1 (24 h after the scratch), and time 2 (48 h after the scratch). For each image, ImageJ (NIH, Bethesda, MD, USA) [58] was used to measure the distances between edges of the scratch.

### 2.7. Statistical Analysis

One-way variance analysis (ANOVA) was used to statistically evaluate the results, which were expressed as the mean ± standard deviation. Student’s t-test was performed considering a two-populations comparison. In the data, *p* < 0.05 was considered statistically significant among groups.

## 3. Results and Discussion

### 3.1. Microstructural Characterization

**Figure 1:**
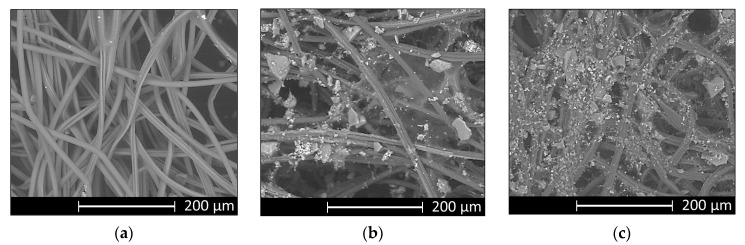
SEM images of microstructure of wound dressings: Chitosan (CHT) (**a**), CHTSrMg5 (**b**), and CHTSrMg10 (**c**), before soaking in simulated body fluid (SBF) solution.

To ascertain that bioactive glass particles were embedded into wound dressings, SEM analyses before immersion in SBF solution were performed. Figure 1 shows that bioactive glass particles were incorporated well into wound dressings.

**Figure 2:**
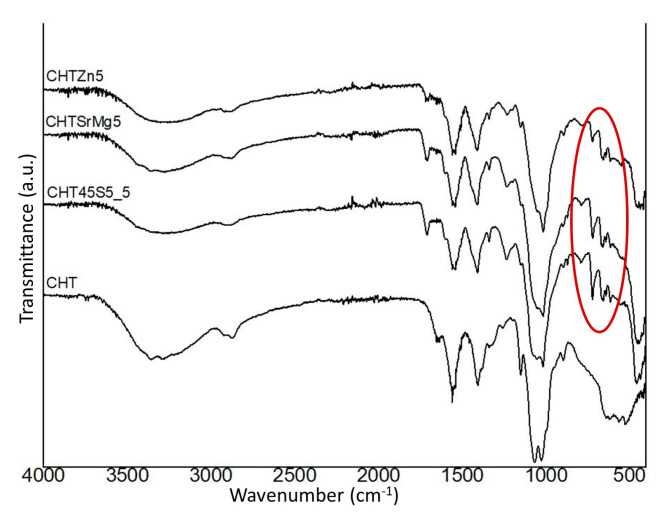
FTIR spectra of CHT, CHT45S5_5, CHTSrMg5, and CHTZn5 wound dressings. Si–O–Si stretching and Si–O–Si bending are highlighted in red.

Furthermore, FTIR spectra of wound dressings containing bioactive glass particles corroborate the good incorporation of bioactive glass particles (Figure 2).

The CHT spectrum shows an intense band around 3400 cm^−1^, which is related to the stretching vibrations of the hydroxyl group [59,60], as well as the N–H groups [61]. Between 2926 and 2876 cm^−1^, symmetric and asymmetric C–H stretching was visible in the spectrum [62]. Other characteristic bands of chitosan were evident at 1063 cm^−1^ (associated with C–O symmetric and asymmetric stretching [63,64]) and at 1156 cm^−1^ (attributed to β-glycosidic bond between the C1 and C4 of chitosan [59]). The band at 1063 cm^−1^ is at a higher wavenumber, while the band at 1156 cm^−1^ is at a lower wavenumber compared to the bands found in [59]. Other bands were observed between 1403 and 1339 cm^−1^ and at 1557 cm^−1^, associated with deformation bands CH_2_ and CH_3_ and the amide, respectively [59,63,65]. Table 3 summarizes the main bands detected by FTIR analysis.

CHT45S5_5, CHTSrMg5, and CHTZn5 spectra show both the characteristic bands of chitosan and bands of Si–O–Si stretching and bending, which are associated with bioactive glasses (Figure 2). With bioactive glass particles embedded into the wound dressing, the characteristic bands of chitosan are shifted at higher or lower wavenumber compared to bands in the CHT spectrum (Figure 2). The CHT45S5_5 spectrum shows the asymmetric stretching of Si–O–Si with the bonded oxygen at 1235 cm^−1^ [17]. Furthermore, other characteristic bands at 1017 cm^−1^ and between 724 and 465 cm^−1^, which are the asymmetric stretching Si–O–Si and bending Si–O–Si of 45S5 particles, respectively, were detected as well [17]. CHTSrMg5 shows the same characteristic bands as the CHT45S5_5 wound dressing, but the bands at 787–461 cm^−1^ associated with Si–O–Si bending are shifted compared to the same bands in the CHT45S5_5 spectra (Figure 2). Again, between 791 and 465 cm^−1^ and 724 and 616 cm^−1^, Si–O–Si bending associated with BGMS_2Zn are visible in the CHTZn5 spectrum (Figure 2).

### 3.2. Swelling Property

**Figure 3:**
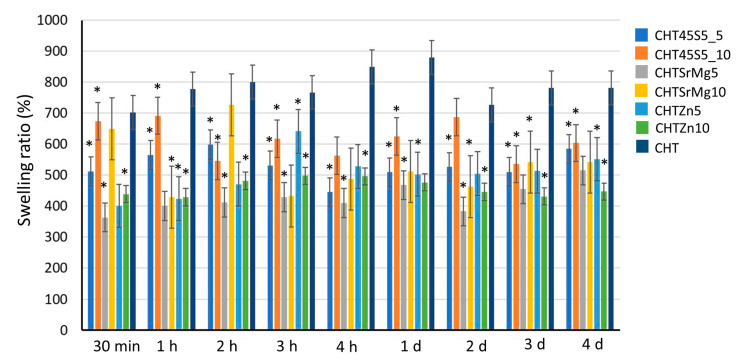
Swelling ratio of wound dressings: 30 min, 1 h, 2 h, 3 h, 4 h, 1 day, 2 days, 3 days, and 4 days after immersion in distilled water. * *p* < 0.05.

The swelling behavior of CHT, CHT45S5_5, CHT45S5_10, CHTSrMg5, CHTSrMg10, CHTZn5, and CHTZn10 in distilled H_2_O was investigated to evaluate the capacity of wound dressings to preserve a wet environment [50,51]. The capacity to retain water is important when wound dressings are applied on an open wound, to absorb exudates, body fluids, and metabolites [66]. The better absorbing capacity of wound dressings keeps the wound dry and prevents air infection [67]. Figure 3 shows the swelling behavior of CHT, CHT45S5_5, CHT45S5_10, CHTSrMg5, CHTSrMg10, CHTZn5, and CHTZn10 in distilled water. Wound dressings with bioactive glass particles show less swelling capacity compared to CHT wound dressings. This behavior could be due to bioactive glass particles, which reduce the wound dressings porosity. A lower swelling ability was expected for wound dressings containing 10 wt.% of bioactive glass particles. However, at each time point, wound dressings containing 10 wt.% of bioactive glass particles do not always show the lowest swelling behavior. Nevertheless, despite the fluctuating trend, the swelling behavior of each wound dressing decreases with increasing time of immersion (*p* < 0.05).

### 3.3. In vitro Bioactivity Investigation

**Figure 4:**
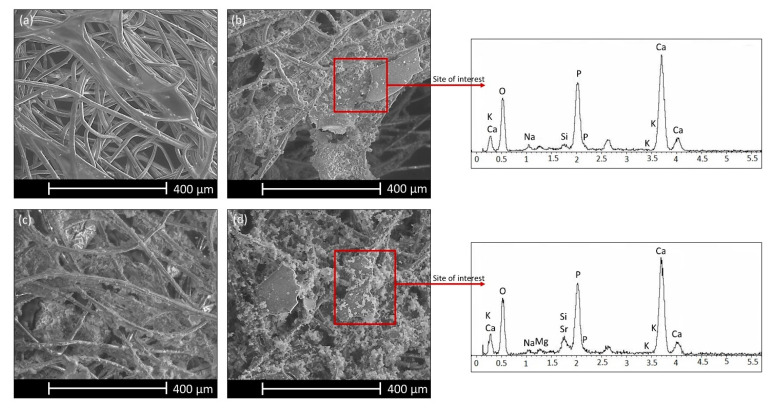
SEM images and EDS spectra 7 days after immersion in SBF solution of CHT (**a**), CHT45S5_10 (**b**), CHTZn10 (**c**), and CHTSrMg10 (**d**) wound dressings.

One, three, and seven days after immersion in SBF, SEM analyses were performed on the surface of CHT, CHT45S5_5, CHT45S5_10, CHTSrMg5, CHTSrMg10, CHTZn5, and CHTZn10. Seven days after immersion in SBF solution, some HCA deposits are visible on CHT45S5_5, CHT45S5_10, CHTSrMg5, and CHTSrMg10 wound dressings. Such deposits are absent on CHT, CHTZn5, and CHTZn10 wound dressings. CHT shows low bioactivity (Figure 4a), which could be improved by the incorporation of bioactive glass particles.

SEM images of CHT45S5_10 and CHTSrMg10 (Figure 4b,d) confirm the presence of HCA deposits especially near bioactive glass particles; this result is corroborated by EDS analysis. Therefore, the incorporation of bioactive glass particles enhances bioactivity, which, in turn, could enhance cell proliferation [68,69]. On the other hand, HCA precipitation was not observed on wound dressings containing BGMS_2Zn (Figure 4c). It is known that Zn ions hinder ions involved in HCA precipitation, reducing the bioactivity of bioactive glasses [70]. Zn ions could form tetrahedral species ZnO_4_^2−^, which bond Ca ions for charge balancing. This removes Ca cations from the silica network, decreasing bioactivity and reducing glass dissolution [71,72]. Even though bioactivity in SBF is not a fundamental feature for wound dressings, such a test was performed in accordance to some papers, which tested the bioactivity on films or hydrogels developed for healing applications [26,73]. In fact, HCA formation may not be exhaustive for predicting the healing ability of CHT, CHT45S5_5, CHT45S5_10, CHTSrMg5, CHTSrMg10, CHTZn5, and CHTZn10 wound dressings in vivo. For this reason, NR uptake, MTT, and the “scratch test” were performed using NIH 3T3 cells.

### 3.4. Biological Investigations

**Figure 5:**
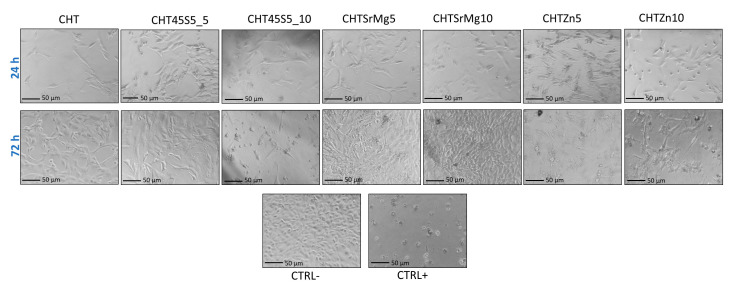
Morphological evaluation of cells (NIH 3T3) 24 and 72 h after seeding in direct contact with wound dressings by optical microscopy (Nikon TMF, Kumagaya, Japan); 50 × magnification.

**Figure 6:**
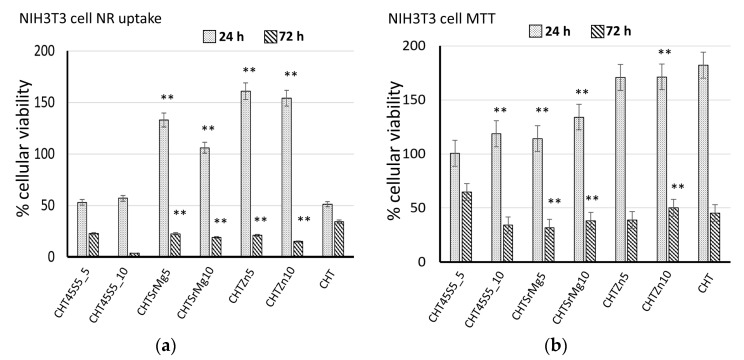
Neutral red (NR) uptake 24 and 72 h after seeding (**a**); MTT 24 and 72 h after NIH 3T3 cultured in eluates from wound dressings (**b**). ** *p* < 0.05.

Biological tests were performed on CHT, CHT45S5_5, CHT45S5_10, CHTSrMg5, CHTSrMg10, CHTZn5, and CHTZn10 to ascertain the biocompatibility of such wound dressings. At 24 and 72 h after direct contact with wound dressings, no significant differences in cellular morphology were observed (Figure 5). The morphology of NIH 3T3 cells on wound dressings is similar to that on CTRL- without showing lysis or rounding. NR uptake and MTT assays confirm results obtained by morphological observation of the cells. Both NR uptake and MTT assays showed higher cellular viability 24 h after seeding (Figure 6). Both 24 and 72 h after direct contact (NR uptake assay), CHTSrMg5, CHTSrMg10, CHTZn5, and CHTZn10 showed higher cellular viability values compared to CHT samples (Figure 6a); in fact, Sr, Mg, and Zn ions are known to promote a specific cellular response, activating molecular signaling involved in the cell cycle [74,75,76,77]. However, 72 h after direct contact, wound dressings showed reduced cellular viability values compared to cellular viability at 24 h after seeding. This could be ascribed to particulates released into the cell medium, which could inhibit cell growing.

Therefore, to avoid any eventual problem caused by particulates released from materials, an MTT assay was carried out (Figure 6b). Then, 24 h after seeding, CHT45S5_10, CHTSrMg5, CHTSrMg10, and CHTZn10 showed higher cellular viability compared to the CHT wound dressing (Figure 6b), confirming results obtained by NR uptake. Next, 72 h after seeding, wound dressings containing bioactive glass particles showed higher cellular viability compared to that shown by the CHT wound dressing. However, it should be noted that the cellular viability of wound dressings at 72 h is lower than that at 24 h; this phenomenon could state the beginning of damaging steps that lead to dead cells. Nevertheless, wound dressings are expected to be changed a few hours after their application on wounds (at least within 24 h); therefore, the beginning of the damage of cells at 72 h after seeding is not particularly concerning. For both NR uptake and MTT assays, CHTSrMg5, CHTSrMg10, CHTZn5, and CHTZn10 wound dressings showed higher cellular viability values compared to other wound dressings. Reasonably, this behavior could be attributed to the release of Zn, Sr, and Mg ions [74,75].

**Figure 7:**
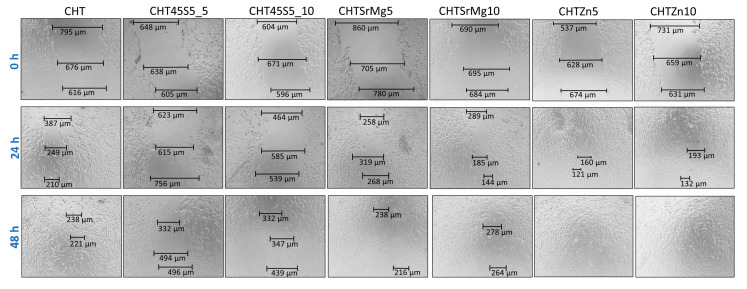
Cell migration using NIH 3T3 in contact with eluates of CHT, CHT45S5_5, CHT45S5_10, CHTSrMg5, CHTSrMg10, CHTZn5, and CHTZn10 wound dressings.

Furthermore, to assess the effect of bioactive glass particles and chitosan on mouse embryonic fibroblasts (NIH 3T3), the closure of the gap in monolayers of mouse embryonic fibroblasts was investigated. Gaps on the monolayer of cells were created by the tip of a 200 μL pipette, and at 24 and 48 h, the closure of gaps was measured. At 24 h, there was a gradual closing defect for all wound dressings (Figure 7).

**Figure 8:**
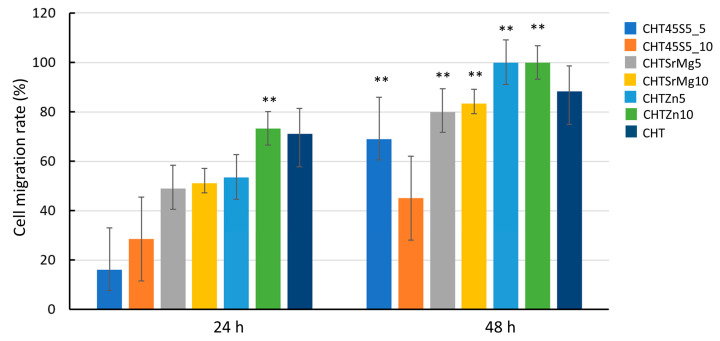
Cell migration rate (%) 24 and 48 h after the “scratch” on confluent monolayer of NIH 3T3 in contact with eluates of CHT, CHT45S5_5, CHT45S5_10, CHTSrMg5, CHTSrMg10, CHTZn5, and CHTZn10 wound dressings. ** *p* < 0.05.

A significant closure in terms of cell migration rate (%) (which can be considered as a measure of the wound healing rate) was registered for CHT and CHTZn10, i.e., 71.06% and 73.31%, respectively (Figure 8).

Thus, at 24 h, CHT and CHTZn10 further narrowed the scratch. At 48 h, the cell migration rate (%) of wound dressings containing bioactive glasses was higher compared to that measured for CHT, and the scratch completely disappeared in CHTZn5 and CHTZn10, and almost disappeared in CHTSrMg10 (Figure 7). In particular, the cell migration rate (%) was significantly higher for CHTZn5 and CHTZn10 (i.e., 100%) compared to the cell migration rate of other wound dressings (Figure 8). The closure of the gap in the scratch of mouse embryonic fibroblasts is more effective in the presence of Zn-containing bioactive glass particles compared to pure CHT and also other wound dressings containing bioactive glass particles (45S5 and BGMS10). The reason could be found in the capacity of chitosan and ions released by different bioactive glasses to activate specific signaling pathways that promote specific cellular responses [78]. As reported in the literature, chitosan can activate the Wnt canonical pathway through the formation of complex Wnt3a, Frizzled, and Lrp5. These complexes increase the level of β-catenin that moves into nuclei, leading to the increase in expression of genes into cells promoting osteogenic differentiation [74]. Chitosan is the main component of all wound dressings even though CHT45S5_5, CHT45S5_10, CHTSrMg5, CHTSrMg10, CHTZn5, and CHTZn10 have 45S5, BGMS10, and BGMS_2Zn bioactive glasses embedded. In the case of chitosan/bioactive glass wound dressings, 45S5 releases Ca and Si ions, which are both involved in HCA layer formation and specific signaling pathways [48,79]. Ca ions play a part in the advancement of the normal cell cycle participating in cell cycle checkpoints. Ca^2+^ signaling affects every aspect of a cell’s life and death, as reported in [75]. Additionally, Ca^2+^ binds to numerous proteins to effect changes in function, localization, and association, and those ions are involved in the regulation of mitochondrial shuttling, Golgi, endosomal and vesicular fusion events, etc. [80]. Besides, Si ions—other than being involved in the formation of a silica layer on samples’ surface before HCA formation—have a strong stimulatory effect on alkaline phosphatase (ALP) activity [81]. Furthermore, Sr ions released by BGMS10 and BGMS_2Zn embedded in other wound dressings (CHTSrMg5, CHTSrMg10, CHTZn5, and CHTZn10) have shown to significantly improve the expression of ang-1 and vascular endothelial growth factor (VEGF) compared to Si ions [81]. The release of Si and Sr ions result in the synergistic effect on osteogenesis and angiogenesis [81]. Additionally, CHTZn5 and CHTZn10 release Zn ions, which stimulate protein synthesis and enhance ALP activity as well, as reported in [82]. The free intracellular Zn level influences the signaling pathway and stability [83] by altering the ligand binding to the receptor, modifying their mutual affinity [84]. The intracellular zinc can activate MAPK/ERK and Akt pathways, which enhance cell proliferation and migration [85]. Furthermore, the release of Zn into the cytoplasm seems to be reactive oxygen species (ROS)-induced. Both the increase in ROS level and the Zn signal are required to subsequently activate key immune functions [86]. Therefore, the high cell migration rate (%) of wound dressings containing bioactive glass particles (Figure 8) could be ascribed to the releasing of ions, which are known to enhance cell adhesion and proliferation by inducing a specific cellular response. In fact, the synergistic effect of Ca, Si, Sr, and Zn ions improves cell proliferation and differentiation, resulting in a higher cell migration rate (%) in the in vitro “scratch assay” [87]. Bioactive glass particles improve wound healing while chitosan shows electrostatic interaction with proteoglycans, anionic glycosaminoglycans (GAG), as well as neutrophils and macrophages, which begin the healing process, and stimulate granulation tissue and re-epithelialization [88,89]. The dissolution of ions (i.e., Ca, Si, Na, Sr, Zn) stimulates angiogenesis in vitro and in vivo and stimulates the expressions of genes related to osteoblastic differentiation and antibacterial and anti-inflammatory actions [82]. Furthermore, Zn is known to have an antibacterial effect [25,41,42], which could help the in vivo healing; more extensive investigations on the antibacterial properties of these wound dressings and, in particular, of CHTZn5 and CHTZn10 deserve to be the topic of future research contributing to support the suitability of these wound dressings.

However, although the addition of bioactive glass particles to chitosan improves the wound healing rate, the bioactive glass particle addition is still far from the challenge of healing chronic wounds.

## 4. Conclusions

Wound dressings are complex systems employed to protect and heal different types of wound. The research is driven by the aim to find innovative wound dressing or healing systems to enhance healing and reduce patient pain. In this work, commercial passive gauzes were immersed in chitosan-based bioactive glass suspension to produce active wound dressings with enhanced cell proliferation and healing. Bioactive glasses of different compositions (45S5, BGMS10 and BGMS_2Zn) were added to the chitosan solution in different amounts (5 wt.% and 10 wt.%, respectively). Bioactive glass could elicit a specific cellular response and could introduce antibacterial properties, enhancing the biological properties of chitosan. Among other features, an ideal wound dressing shows the capacity to preserve high humidity while removing excess exudates; for this reason, the swelling test was performed. Wound dressings containing bioactive glass particles showed a lower swelling capacity compared to chitosan wound dressings because bioactive glass particles inhibit the capacity of chitosan to absorb exudates. On the other hand, bioactive glass particles enhance chitosan bioactivity because HCA precipitate was observed 7 days after immersion in SBF solution on wound dressings with bioactive glass particles only. Therefore, wound dressings with bioactive glass particles showed a higher bioactivity compared to wound dressings with chitosan. Furthermore, to exclude cytotoxicity, NR uptake and MTT assays using NIH 3T3 cells were carried out. Furthermore, once again, 24 and 72 h after seeding, wound dressings with bioactive glass particles showed higher cellular viability values compared to the cellular viability of chitosan wound dressings; this could be ascribed to the ions released by bioactive glass particles. Finally, the in vitro scratch test showed better migration of cells for wound dressings containing BGMS10 and BGMS_2Zn compared to wound dressings containing 45S5 and to chitosan wound dressings. In the case of CHTZn5 and CHTZn10, the cellular gap was almost already closed 24 h after the scratch, and 48 h after the scratch, the gap was completely closed. Then, 48 h after the scratch, for CHTSrMg5 and CHTSrMg10, the gap was closed, while for other wound dressings, the gap was still visible. The release of Sr, Mg, and Zn ions improved cell proliferation and wound healing rate. Therefore, wound dressings containing BGMS10 and BGMS_2Zn showed favorable properties for healing, and this makes them suitable materials for in vivo studies. Thus, wound dressings with bioactive glass particles could represent a starting composite for biomedical application, even if a better comprehension of molecular and biological response is still needed.

## Figures and Tables

**Table 1:** Composition of 45S5 and the produced bioactive glasses.

Oxides	Composition (mol%)		
	45S5	BGMS10 [24]	BGMS_2Zn [25]
**SiO_2_**	45	47.2	47.2
**P_2_O_5_**	6	2.6	2.6
**Na_2_O**	24.5	2.3	2.3
**K_2_O**	0	2.3	2.3
**CaO**	24.5	25.6	25.6
**MgO**	0	10	8
**SrO**	0	10	10
**ZnO**	0	0	2

**Table 2:** Amount of materials and sample names.

Amount (wt.%/Vtot)							
	CHT	CHT45S5_5	CHT45S5_10	CHTSrMg5	CHTSrMg10	CHTZn5	CHTZn10
**Chitosan**	1	1	1	1	1	1	1
**Acetic Acid**	2	2	2	2	2	2	2
**45S5**	0	5	10	0	0	0	0
**BGMS10** [24]	0	0	0	5	10	0	0
**BGMS_2Zn** [25]	0	0	0	0	0	5	10

**Table 3:** Overview of the main bands detected in FTIR spectra of wound dressings.

Wavenumber (cm^−1^)	CHT45S5_5	CHTSrMg5	CHTZn5	Assignment	Abbreviation	Ref.
3366–3295		3307	3291	Overlapping OH & NH stretching vibration	υ (OH & NH)	[59]
2926–2876	2886	2931-2868	2923–2881	Symmetric and asymmetric stretching C–H	υ_as_ & υ_s_ (C–H)	[62]
1063	1055	1051	1055	C–O symmetric and asymmetric stretching	υ_s_ & υ_as_ (C–O)	[63,64]
1156	1151	1151	1151	Stretching β-glycosidic bond between the carbon 1 and 4 of chitosan	υ C 1 & C 4	[59]
1403–1339				Deformation of CH_2_ & CH_3_	δ CH_2_ & CH_3_	[59,63]
1557	1557	1553	1541	Amide		[63]
/	1231	1235	1235	Asymmetric stretching Si–O–Si with bonded oxygen	υ_as_ Si–O–Si (BO)	[17]
/	1017	1017	1017	Asymmetric stretching Si–O–Si	υ_as_ Si–O–Si	[17]
/	724–465	721–451	791–465	Symmetric stretching & bending Si–O–Si	υ_s_ Si–O–Si & δ Si–O–Si	[17]
